# ^18^F-FDG PET scanning of abdominal aortic aneurysms and correlation with molecular characteristics: a systematic review

**DOI:** 10.1186/s13550-015-0153-8

**Published:** 2015-12-23

**Authors:** U. T. Timur, J. A. van Herwaarden, D. Mihajlovic, P. De Jong, W. Mali, F. L. Moll

**Affiliations:** Department of Vascular Surgery, UMC Utrecht, Heidelberglaan 100, Utrecht, 3584 CX Netherlands; Deparment of Radiology, UMC Utrecht, Heidelberglaan 100, Utrecht, 3584 CX Netherlands

**Keywords:** Aortic aneurysm, AAA, ^18^F-FDG, PET scanning, Rupture risk prediction, Molecular characteristics

## Abstract

**Purpose:**

The purpose of this study is to give an overview of studies investigating the role of fludeoxyglucose F18 (^18^F-FDG) positron emission tomography (PET) scanning in patients with aortic aneurysms with a focus on molecular characteristics of the aneurysm wall.

**Methods:**

MEDLINE, EMBASE, and the Cochrane database were searched for relevant articles. After inclusion and exclusion, we selected 18 relevant articles reporting on ^18^F-FDG PET scanning of aortic aneurysms.

**Results:**

The sample size of studies is limited, and there are no standardized imaging protocols and quantification methods. ^18^F-FDG PET scanning was shown to display molecular characteristics of the aortic wall. Different studies showed contradictory findings of aortic ^18^F-FDG uptake in aneurysm patients compared to controls.

**Conclusions:**

Non-invasively determining molecular characteristics of aortic wall weakening might lead to better rupture and growth prediction. This might influence the decision of the surgeon between conservative and surgical treatment of aneurysms. To date, there is conflicted evidence regarding the use of ^18^F-FDG PET scanning to predict aneurysm rupture and growth. The role of ^18^F-FDG PET scanning in rupture risk prediction needs to be further investigated, and standardized imaging protocols and quantification methods need to be implemented.

**Electronic supplementary material:**

The online version of this article (doi:10.1186/s13550-015-0153-8) contains supplementary material, which is available to authorized users.

## Review

### Introduction

Abdominal aortic aneurysm (AAA) is an abnormal focal dilation of the aortic wall and the most accepted definition for it is a diameter of 3.0 cm or more. Ruptured AAA is a serious complication with an overall mortality rate of 90 %, making it essential to develop strategies to predict rupture. Currently, the decision between conservative versus surgical treatment involves weighing the risk of aneurysm rupture versus the risks of a surgical procedure. This calculation of aneurysm rupture risk is based on assumptions of population-averaged properties for the aneurysm wall based on maximum aneurysm diameter. Anatomic characteristics like aortic tortuosity and diameter asymmetry have also been described as reflectors for rupture risk [1]. However, not only large aneurysms but also small aneurysms can rupture, making the diameter of AAA alone not the ideal determinant in risk stratification [2, 3].

The etiology of AAA is multifactorial including genetic factors. AAA rupture represents a mechanical failure [4], attributable to alterations in extracellular matrix components of the aortic wall. Increased activity of the so-called matrix metalloproteinases, enzymes with proteolytic activity that also play a role in other degenerative diseases like osteoarthritis, has been demonstrated [5–8]. Production of these enzymes by inflammatory cells such as macrophages, B- and T-lymphocytes, and mast cells has been shown [6, 9]. Fludeoxyglucose F18 (^18^F-FDG) is a positron emission tomography (PET) tracer, which is believed to reflect glucose accumulation by inflammatory cells, and thus, it could be useful in non-invasively displaying inflammatory characteristics of the aneurysm wall. It would be of great importance, both from the patient’s perspective and as from a health-care economical point of view, to predict aneurysm rupture by non-invasively detecting inflammatory activity in the aortic aneurismal wall. We therefore conducted a systematic review of human studies in which ^18^F-FDG PET scanning is performed on patients with aortic aneurysms and correlation between ^18^F-FDG PET scanning and clinical events or histology/molecular characteristics is addressed.

### Methods

#### Search strategy

**Fig. 1:**
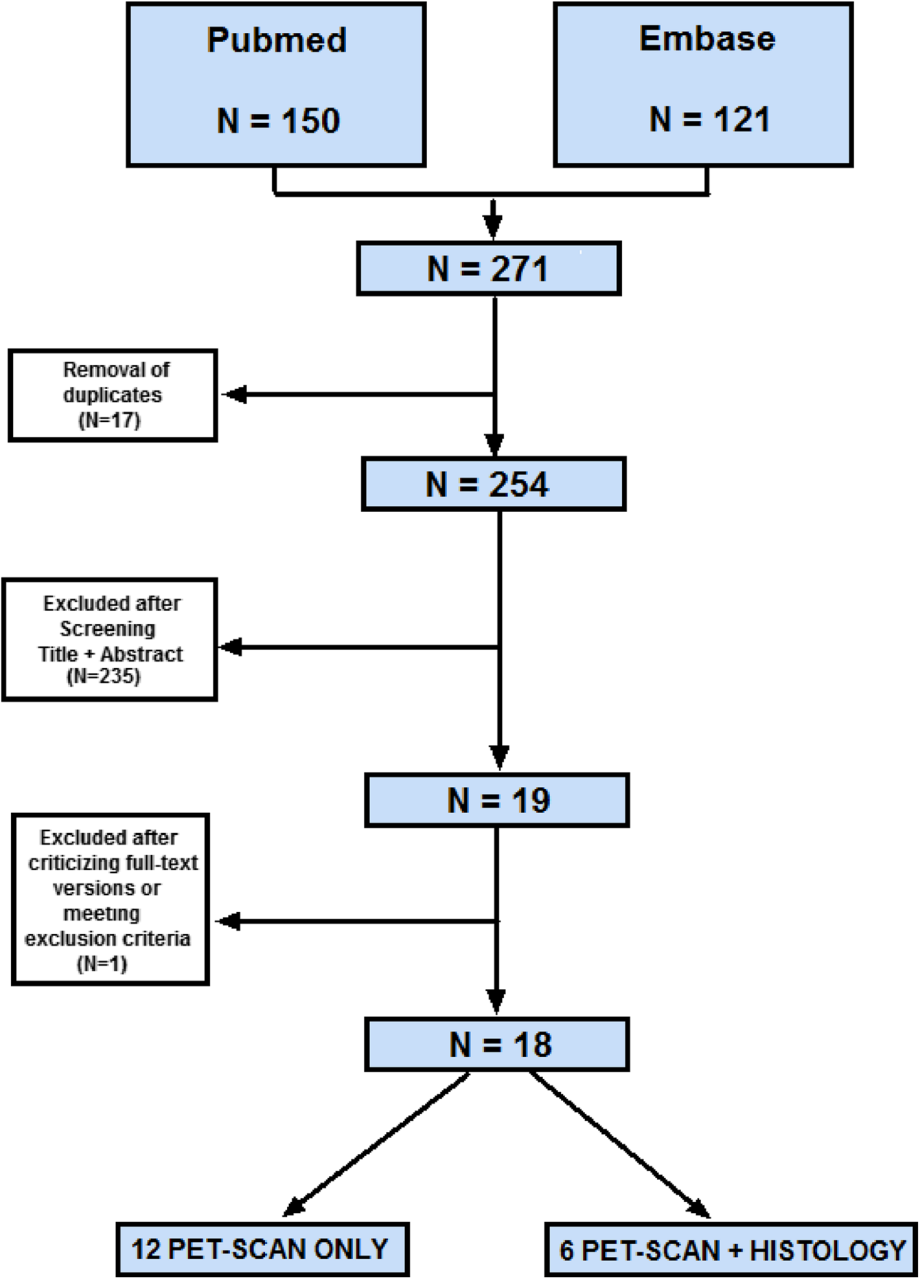
Flowchart of the systematic review

MEDLINE and EMBASE databases were systematically searched on all studies relating abdominal or thoracic aortic aneurysm, ^18^F-FDG PET scanning, and surgically derived aortic wall material (Fig. 1). The search was conducted in September 2015 according to the search strategy and data collection guidelines of the Preferred Reporting Items for Systematic Reviews and Meta-analyses (PRISMA) statement [10]. A manual search of the Cochrane Library yielded no relevant articles.

#### Data collection and extraction

After disregarding duplicates, the title and the abstract of 271 articles were independently screened by two observers (U.T. and D.M) according to predefined criteria. The search query can be found in Additional file 1. Inclusion criteria were as follows: (1) presenting data about patients with thoracic or abdominal aortic aneurysm and (2) ^18^F-FDG PET scanning with or without reporting data on correlation between ^18^F-FDG PET scanning and molecular characteristics. Letters, comments, abstracts for conferences, case reports <10, and animal studies were eliminated. We excluded studies with acute aortic syndromes, as these are generally considered as a separate pathology.

Nineteen articles of studies that matched the inclusion criteria were obtained. Articles were excluded if one of the following criteria were applicable: (1) ex vivo imaging and (2) not written in English. After reading the full text of the articles, we excluded one article because of not focusing on PET of the aortic wall. Reference lists of the included articles were searched manually and yielded no new articles. Disagreements between the reviewers were resolved by consensus.

### Results

Our search resulted in 18 articles in which ^18^F-FDG PET scanning is reported on patients with aortic aneurysms. PET scanning protocols were specified in all studies. QPCR, histology, and immunohistochemistry protocols were described in all studies. PET scanning was either performed in asymptomatic patients or in symptomatic patients, which is in some studies defined by abdominal or lower back pain and in other studies by accelerated growth, leaking, or rupture of the aneurysm. An overview of AAA patients investigated with ^18^F-FDG PET scanning is given in Table 1.

**Table 1:** An overview of studies in patients with AAAs which are investigated with ^18^F-FDG PET scanning

Research group	No. of patients	Mean age in years	Mean AAA diameter in mm	Time of scanning after FDG	Method of PET analysis	Correlation of PET uptake with
Sakalihasan et al. [11]	26	72	63	60	Visual	Growth, symptoms, leaking and rupture
Reeps et al. [14]	12	69	59	90	SUV_max_	AAA disease, symptoms, histopathologic characteristics of aneurysm wall instability
Truijers et al. [13]	17	71	40	60	SUV_max_	AAA disease
Kotze et al. [20]	14	73.6	54	180	(SUV_max)_	–
Menezes et al. [27]	17	74	53	45, 60, 120, 180	TBR (SUV_max_)	–
Kotze et al. [22]	25	75	50	180	TBR (SUV_max_)	Inverse correlation between baseline FDG uptake and future aneurysm expansion
Muzaffar et al. [28]	15	74	50	60	SUV_max_	–
Marini et al. [17]	12	73	48	60–90	TBR (SUV_max_)	Lower FDG uptake in AAA patients compared to controls
Palombo et al. [16]	40	74	49	≥60	Visual, TBR (SUV_max_)	Lower FDG uptake in AAA patients compared to controls
Tegler et al. [15]	12	65	58	60	SUV_max_,	–
Reeps et al. [24]	18	71	56	90	SUV_max_, SUV_mean_	Symptoms, histopathologic characteristics of aneurysm wall instability
Courtois et al. [25]	8 PET+	78	54.5	60	TBR (SUV_max_)	Growth, symptoms, histopathologic characteristics of aneurysm wall instability
	10 PET−	75	58.5	60		–
Courtois et al. [26]	6 PET+	79	54.5	60	TBR (SUV_max_)	Histopathologic characteristics of aneurysm wall
	6 PET−	72	56.5	60		
Kotze et al. [23]	40	75	48.5	180	TBR (SUV_max_)	Negative correlation with aneurysm expansion
Nchimi et al. [12]	53 (6 TAA)	72	41.7	54–100	TBR (SUV_max_)	Future clinical events, wall stress
Barwick et al. [19]	151	74	50	90	TBR (SUV_max_)	Lower SUV_max_ in AAA patients compared to controls
Morbelli et al. [18]	30	74.6	49	90	TBR (SUV_max_)	Lower FDG uptake in AAA patients compared to controls
Morel et al. [21]	39	71	46	90	TBR (SUV_max_)	Inverse correlation between baseline FDG uptake and future aneurysm expansion

TBR = Target to Background Ratio

#### Articles correlating ^18^F-FDG uptake in AAA patients to clinical events

In 2002, Sakalihasan et al. were the first to report the correlation of ^18^F-FDG uptake in AAA patients to clinical events. In this study, 26 patients with AAA were included and visual uptake of ^18^F-FDG was seen in ten patients [11]. Patients with asymptomatic AAA but also patients presenting with lower back pain and AAA (symptomatic AAA) were analyzed. While patients with negative ^18^F-FDG uptake required no urgent surgery, five of the ten patients with positive uptake required urgent surgery within 2 to 30 days. However, not all symptomatic AAAs showed ^18^F-FDG uptake. Next to the study performed by Sakalihasan et al., another study correlated ^18^F-FDG uptake to clinical events in AAA patients. Nchimi et al. studied 53 patients with aortic aneurysms, of which 6 are with thoracic aortic aneurysms [12]. More clinical events (rupture, dissection, or growth >1 cm) occurred in patients with visually increased ^18^F-FDG uptake. Quantitatively, ^18^F-FDG positron emission tomographic uptake correlated positively with both wall stress and stress/strength index.

#### Articles comparing ^18^F-FDG uptake between AAA patients and controls without AAA disease

Findings published by Sakalihasan et al. in 2002 prompted further research to investigate the role of ^18^F-FDG PET scanning in rupture risk prediction. Several studies have been performed in which ^18^F-FDG uptake of AAA patients is compared to ^18^F-FDG uptake in controls without AAA disease. In a study published in 2008 by Truijers et al., 17 patients with asymptomatic AAA were investigated retrospectively and maximum standardized uptake values (SUV_max_) were compared to age-matched controls. ^18^F-FDG PET scanning in both the patient and control groups was performed for staging of primary lung cancer [13]. Patients had significantly higher SUV_max_ values than controls. Accordingly, Reeps et al. report increased SUV_max_ values in 12 patients with asymptomatic AAA compared to an age-matched control group without aortic aneurysm disease [14]. In 2012, Tegler et al. [15] examined seven asymptomatic men with large AAA (range, 52–66 mm) and five asymptomatic men with small AAA (range 34–40 mm) with ^18^F-FDG PET scanning. Consistent with the findings of Truijers et al. and Reeps et al., a significant increase in SUV_max_ was found in asymptomatic patients with AAAs compared to controls without aneurysm [14].

Nonetheless, studies have been published reporting no difference or even decreased ^18^F-FDG uptake in AAA patients compared to controls without AAA disease. In a case-control study conducted in 2012, Palombo et al. compared ^18^F-FDG uptake in aortic walls of 40 male patients with asymptomatic AAA disease to controls (with neoplastic disease) without any clinical evidence for atherosclerotic disease [16]. Patients with AAAs both had lower mean SUV and maximum SUV compared to adjacent non-aneurysmal segments within the same patient but also compared to controls. Consistent with the findings of Palombo et al. [16], another study published by Marini et al. in the same year reports decreased SUV_max_ in the aneurismal walls of 12 patients with asymptomatic AAA, compared to 12 age- and sex-matched controls (with neoplastic disease) [17]. Morbelli et al. confirmed findings of Palombo et al. [16] and Marini et al. [17]. In this study, ^18^F-FDG uptake in 30 AAA patients was compared to 30 controls. Decreased ^18^F-FDG uptake was seen in the aneurysms of AAA patients in comparison to the corresponding arterial segment of the control group but also in comparison to the non-aneurysmal segment of the same patient [18]. Another case-control study is published by Barwick et al. in 2014 [19]. They searched a PET/CT database of predominantly oncological patients and matched 151 aneurysm patients to 159 non-aneurysmal controls and do not report significant differences in visual ^18^F-FDG uptake or SUV_max_ between patients and controls. Moreover, no significant differences were found in SUV_max_ between patients who underwent surgery, had AAA rupture, or did not have rupture or surgery.

#### Articles correlating ^18^F-FDG uptake to aneurysm expansion

Since AAA has an inflammatory component, increased inflammatory activity in the aortic wall could potentially indicate recent growth of an aneurysm and thus help in rupture risk prediction. Two studies investigated the correlation between ^18^F-FDG uptake and recent AAA growth [14, 20], yet no correlations were found. In addition, ^18^F-FDG uptake in the aortic aneurismal walls may indicate future aneurysm expansion. Three studies investigated the relationship between ^18^F-FDG uptake and future expansion [21-23]. Kotze et al. investigated ^18^F-FDG uptake in 25 AAA patients with small aortic abdominal aneurysms and measured aneurysm expansion rate 6 months and 1 year later with ultrasound. Of the 25 patients included, three patients had lower back or abdominal pain. A significant inverse correlation was found between whole-vessel SUV_max_ and ultrasound expansion at 1 year after scanning [22]. This research group again reported the same findings in 40 AAA patients, of which 2 presented with lower back pain [20]. SUV_max_ again correlated inversely with further aortic expansion at 1 year measured by ultrasound.

Recently, an article reported about the potential of ^18^F-FDG in risk stratification of AAA [21]. In this study, patients with AAA <55 mm underwent ^18^F-FDG PET scanning at baseline and 9 months later. Patients with an increase in AAA size after 9 months had significantly lower ^18^F-FDG uptake at baseline compared to patients without significant increase in AAA size. Moreover, the increase in ^18^F-FDG uptake throughout time was higher in patients displaying a significant increase in AAA size.

#### Correlation of ^18^F-FDG uptake to histology of the aortic wall in AAA

The ability of ^18^F-FDG PET scanning to non-invasively detect histopathological characteristics of the aneurismal wall was investigated in six studies (Table 1).

Reeps et al. [14] studied 12 asymptomatic and 3 patients presenting with aneurysm-specific abdominal pain: symptomatic AAA. All patients underwent ^18^F-FDG PET/CT, followed by open AAA repair. Analysis by immunohistology was done from areas with maximum ^18^F-FDG uptake. Immunohistological analysis showed that increasing SUV_max_ levels were significantly associated with increasing medial inflammatory cell infiltrates, higher densities of CD68-positive macrophages, and with CD3-positive T-lymphocytes. Moreover, increased ^18^F-FDG uptake was significantly associated with increased MMP-9 expression. Furthermore, significant negative correlation of collagen fiber and vascular smooth muscle cells (VSMC) content compared with increasing SUV_max_ was found_._ Five years later, the same research group also investigated the role of partial volume correction in accurate quantitative assessment of ^18^F-FDG uptake in the same patient group [24]. Partial volume corrected mean SUV (PVC-SUV_mean_) and maximum SUV (PVC-SUV_max_) were determined. Both PVC-SUV_mean_ and PVC-SUV_max_ were significantly higher than the uncorrected SUV_mean_ and SUV_max._ Previously demonstrated significance correlation of ^18^F-FDG uptake with macrophage infiltration and increased MMP-9 expression did not change by applying partial volume correction nor improved correlation coefficients.

In 2012, Tegler et al. [15] investigated seven patients with large AAAs (size 52–66 mm) and five patients with small AAAs (size 30–40 mm). No visual uptake of ^18^F-FDG was seen, while histological analysis of specimens taken from the aneurismal wall of the seven patients with large AAAs all showed high inflammatory cell infiltration with B-lymphocytes, T-lymphocytes, and macrophages.

Marini et al. report a decrease in cell density in aneurysmal wall biopsies of AAA patients. They concluded that, because a significant relation was found between cell density and ^18^F-FDG uptake, reduced cell density in these patients account for the low prevalence of positive findings of AAA patients at PET imaging [17].

In a study published in 2013 by Courtois et al. [25], PET/CT imaging in 18 patients with symptomatic or asymptomatic AAA was performed. Eight of the patients showed ^18^F-FDG uptake (PET+), while ten showed no ^18^F-FDG uptake (PET−). A comparison was made in immunohistology, mRNA, and protein levels of PET+ and PET− patients. Moreover, biopsies of the AAA wall in regions with maximum ^18^F-FDG uptake were also compared to biopsies in the same patient where no uptake of ^18^F-FDG was seen. No significant correlation was found between AAA diameter and ^18^F-FDG uptake. Significantly higher levels of circulating C-reactive protein (CRP) were found preoperative in PET+ patients. Inflammatory infiltrate in the adventitia was significantly higher in the PET+ group compared to the PET− group or the biopsy taken from the negative site. The density of smooth muscle cells in the media was significantly reduced in the positive ^18^F-FDG uptake sites as compared with their respective negative counterparts and with the PET− patients. The mRNA and protein levels of extracellular matrix degrading enzymes (MMPs) in the media but also adventitia significantly increased in ^18^F-FDG positive sites, compared to negative sites in the same patients.

A similar approach was used again by the same research group very recently, in which 12 AAA patients were included, with six of the patients showing ^18^F-FDG uptake and six showing no uptake. Regions with ^18^F-FDG uptake showed increased gene expression levels of markers involved in inflammatory processes and extracellular matrix remodeling. Moreover, increased levels of a chemokine, CCL18, were found in the adventitia of patients with ^18^F-FDG uptake [26].

#### Articles reporting solely PET scanning in AAA patients without correlations

Some studies solely investigated ^18^F-FDG PET scanning without correlating it to clinical events, aneurismal growth, or histological characteristics. In a study, performed in 2009 by Kotze et al., 14 patients with AAA were investigated of which one presented with lower back pain [20]. Twelve of these patients showed increased ^18^F-FDG uptake, defined by this group by a SUV_max_ >2.5 but not correlations were made.

Menezes et al. examined 17 patients with asymptomatic AAA and performed PET scans at several time points after ^18^F-FDG injection [27]. They conclude that there is no significant advantage in imaging 3 h over 1 h after ^18^F-FDG injection. In this study, no correlation of PET scanning to clinical characteristics is reported. In 2011, Muzaffar et al. reviewed ^18^F-FDG PET/CT scans from 926 patients with cancer and found AAA in 15 patients [28]. This study solely reports a SUV_max_, without correlating it to clinical characteristics.

### Discussion

In this article, we give an overview of what is known about ^18^F-FDG PET scanning in patients with aortic aneurysms. Only one study reports inclusion of patients with thoracic aneurysms [12]. Imaging modalities as MRI and CT scanning were excluded, because an earlier search yielded no useful articles to discuss in this review.

^18^F-FDG PET scanning is an evolving imaging tool in the evaluation of inflammatory disorders and might thus be useful in predicting rupture risk. Indeed, Xu et al. [29] showed that high wall stress regions, calculated using the finite element method, colocalize with areas of positive ^18^F-FDG uptake. These results are consistent with the findings of Nchimi et al. [12].

Contradictory reports on ^18^F-FDG uptake in AAA patients compared to controls can be found in this review. Patient selection is a possible explanation for these contradictions. For instance, Sakalihasan et al. scanned large, rapidly expanding or symptomatic AAAs [11]. In addition, some studies scanned patients prior to surgery [14–17, 25, 26], while other studies analyzed ^18^F-FDG PET scans of AAA patients under routine surveillance, either prospectively [12, 21–23] or retrospectively [13, 19]. Furthermore, studies investigated both patients and controls with a neoplastic disease [13, 19], but studies also report on ^18^F-FDG PET scanning in AAA patients without neoplastic disease and compare this to a control group with neoplastic disease [16, 17]. As neoplasms display increased ^18^F-FDG uptake, this might lead to false positive readings. Moreover, two studies did not specify the reason of ^18^F-FDG PET scanning in their control group [14, 15].

^18^F-FDG uptake in AAA patients should be compared to a control group without atherosclerosis: patients without hypertension, hyperlipidemia, and non-smokers. Atherosclerosis is a systemic disease, and results in the control group might be influenced by calcification. While several studies described their control group [13, 16, 17, 19], others did not [14, 15]. Currently, not much is known about calcification and ^18^F-FDG uptake. There are some reports in the literature suggesting that ^18^F-FDG uptake precedes calcification [30, 31]. A study reported congruent ^18^F-FDG uptake with calcification spots on CT in 7 % of calcifications [32], while there is also a study reporting ^18^F-FDG uptake in the thoracic aortic wall, distinct from calcification sites at CT [33]. Rominger et al. retrospectively evaluated 932 patients with ^18^F-FDG PET/CT and show significant correlation between ^18^F-FDG uptake and calcifications in the abdominal aorta [34]. Moreover, increased ^18^F-FDG uptake and increased calcifications in the arterial system were both established as independent predictors for future vascular events, while both increased ^18^F-FDG uptake and calcification were identified as being at the highest risk for a vascular event. Four studies in this review investigated whether there was a difference in calcification between AAA patients and controls. While Kotze et al. [20] and Marini et al. [17] find no significant differences, Palombo et al. [16] and Morbelli et al. [18] report increased arterial calcium load in AAA patients compared to controls. Moreover, an inverse correlation between arterial calcium load and arterial wall metabolism was found.

In addition to patient selection, and incorrect control groups, timing of PET imaging and quantification methods is a possible explanation to contradictory reports in literature. Considerable differences exist in timing of imaging and quantification methodology as reported in studies. Only seven studies specified whether visual uptake of ^18^F-FDG was seen [11, 12, 15, 16, 19, 25, 26]. Some studies describe the use of SUV_max_ or SUV_mean_ divided by blood pool or liver activity [12, 16–19, 21–23, 25–27], while others only use SUV_max_ or SUV_mean_ without blood pool correction [13–15, 20, 24, 28]. This makes it difficult to compare results in literature, highlighting the importance of standardized techniques and quantification methods. Six studies scanned 60 min after ^18^F-FDG injection [11–13, 15, 25, 26, 28], four other studies 90 min [14, 18, 21, 24], and three others [20, 22, 23] 180 min after ^18^F-FDG administration. Blomberg et al. showed improvement in atherosclerotic plaque quantification in the carotid arteries and thoracic aorta scanning 180 min after ^18^F-FDG administration compared to 90 min [35]. However, Menezes et al. [21] show that there is no significant difference in SUV_max_ uptake at 60 min compared to scanning at 180 min.

Correlations between ^18^F-FDG uptake and pathological weakening of the wall can aid in investigating how effective this imaging tool will be in rupture prediction. Correlation between ^18^F-FDG PET and histology was first shown in a case report [36], where ^18^F-FDG uptake corresponded to an inflammatory infiltrate in the aortic wall. In this review, several studies show correlation between ^18^F-FDG uptake and histological aneurysm characteristics. Reeps et al. [14] showed that there is a significant correlation between total inflammatory infiltrate and MMP-9. MMP-9 already has been shown to be significantly upregulated in ruptured sites of AAAs compared to non-ruptured sites [37]. Moreover, its expression is shown to be decreased in non-ruptured abdominal aneurysms compared to ruptured abdominal aneurysms [38]. It remains the question whether the inflammatory infiltrate in the AAA wall is an etiological factor responsible for the increase in MMP or merely a reaction to an unknown etiological factor causing this increase in MMP expression.

Remarkably, Truijers et al. [13], showed the highest ^18^F-FDG uptake in patients with relatively small AAAs, while the patient with the largest AAA showed very low ^18^F-FDG uptake. Moreover, studies that compared ^18^F-FDG uptake in AAA compared to a matched control group reported lower ^18^F-FDG uptake [16–18]. These observations are most likely the result of a reduction in cell density occurring in large AAAs as documented by Marini et al. [17]. In contrast to the aneurysmal segment, the arterial tree of patients with AAA display higher ^18^F-FDG uptake [18]. The dispersed nature of inflammatory cell islands in larger AAAs causes an underestimation of radioactivity concentration whenever the thickness of the source is less than twice the system spatial resolution. PET scanning has limited spatial resolution, and therefore, it remains a challenge to investigate the arterial wall. While findings of Marini et al. do not label ^18^F-FDG PET scanning as an inadequate tool for risk stratification in AAA, it is essential to realize that currently, a patient with a negative PET scan should not be considered as low risk for rupture.

Courtois et al. [25, 26] compared patients with and without ^18^F-FDG uptake as assessed visually. Interesting results are shown that give insight into the pathophysiology of abdominal aortic aneurysms. The significant reduction in expression of MMP-12 and MMP-15 from regions with no ^18^F-FDG uptake in PET+ patients may be indicative of a final attempt in the tissue to restore extracellular matrix. This response might be a futile attempt to protect from a yet unknown etiologic factor, leading to more inflammation and thus to a PET+ patient. However, when analyzing remodeling of ECM, it is important to take into account substrates of proteolytic enzymes [39] and the contribution of the structural protein to tensile strength of the aortic wall.

In humans, ^18^F-FDG is the most frequently used PET tracer in nuclear investigations of aortic aneurysms and was also shown to have the highest sensitivity in a rat experimental AAA model, compared to two other PET tracers involved in leukocyte activation [40]. Tegler et al. investigated two other PET tracers targeting proteins involved in chronic inflammation but were not able to show differences in uptake between AAA patients and controls [41].

Developing effective PET tracers to improve AAA rupture risk stratification should focus on pathophysiological processes in AAA. As AAA walls display a large infiltration of immune cells such as macrophages, PET tracers targeting receptors on macrophages such as integrin αvβ3 might be useful [42]. The search for novel PET tracers that can be useful in predicting AAA rupture is ongoing. Animal studies are helpful in investigating novel molecular probes that might be useful in predicting AAA rupture. English et al. used a novel abdominal aortic aneurysm model in rats and showed that increased ^18^F-FDG uptake is predictive of rupture [43]. Nahrendorf et al. [44] show, by using macrophage-targeted nanoparticles labeled with fluorine-18 in PET/CT scanning, that macrophages localized in the aneurysmal wall can be visualized. Recently, Shi et al. showed angiogenesis in AAA experimental mice by PET scanning with a (64)Cu-labeled anti-CD105 antibody [45]. Also, other imaging techniques are being used for rupture prediction in animal models such as non-invasive MR imaging and near-infrared fluorescence [46, 47]. Future experiments need to prove the ability to use these techniques for rupture risk stratification in AAA patients.

In addition to the aneurysmal wall, improved molecular imaging of the intraluminal thrombus (ILT) not only qualitatively but also quantitatively might provide valuable information helpful in predicting rupture risk. Koole et al. [48] showed that ILT thickness is associated with higher MMP levels and lower vascular smooth cell numbers, which might implicate that AAA wall adjacent to a thick layer of ILT is significantly weaker than wall in the same AAA adjacent to a thinner or no ILT. Moreover, Nchimi et al. showed that the occurrence of ILT precedes AAA peak growth [49].

Symptoms in AAA patients point out to an increased rupture risk, but these indicators of increased risk for AAA rupture are not available in asymptomatic AAA patients. In this patient group, there is a need to establish risk percentages for AAA rupture. Studies report subtle differences in ^18^F-FDG uptake between patients and controls [14, 15, 18, 50]. As definition of quantitative cutoff values is essential in establishing risk percentages for aneurysm rupture and keeping this little difference in SUV_max_ in mind between AAA patients and controls, it is essential to correct for partial volume effects observed in the thin aortic wall [24]. However, in the end, it is unlikely that one single PET scan with a random PET tracer will lead to a reliable single quantitative cutoff value for rupture risk prediction. Serial imaging of AAAs will increase the chance to detect inflammatory activity in the aortic aneurismal wall. Investigating the aneurysm wall metabolism at more than a single time point will give valuable information as formation and expansion of aortic aneurysms takes many years. It is currently unknown whether inflammatory processes lead to expansion of aneurysms. No significant correlation was found between the degree of ^18^F-FDG uptake and recent AAA growth rate or maximum infrarenal AAA diameter in four studies [13, 14, 16, 22]. This supports the hypothesis that inflammation precedes expansion instead of expansion preceding inflammation. However, in contradiction with this hypothesis, Kotze et al. report inverse correlation between whole-vessel ^18^F-FDG uptake and aneurysm expansion at ultrasound after 1 year, indicating that aortic aneurysms with lower metabolic activity may be more likely to expand [22]. AAA formation and progression is a dynamic process, with repetitive sequences of inflammatory damage and repair. Periods of rapid expansion are followed by periods of quiescence [51]. It is likely that this dynamic process causes a cyclic variation in ^18^F-FDG uptake. Indeed, findings published by Morel et al. support evidence of cyclic changes in the metabolism of AAA during growth phases [21]. Consistent with findings published by Kotze et al. [22], AAAs with lower ^18^F-FDG uptake were more likely to expand in this study. It is likely that aneurysms with lower ^18^F-FDG are at the end of their “period of stasis” and will start with their “period of expansion.” Next to the study published by Morel et al. [21], solely one case study reports a correlation between aneurysm wall glucose metabolism and inflammatory changes with an increase in SUV_max_ and aneurysm size over time [52]. Following AAA patients with repeated PET/CT scans might be useful, but this approach needs to be weighed against higher radiation exposure.

## Conclusions

Currently, a limited number of studies have investigated the role of ^18^F-FDG PET scanning in patients with AAAs and the correlation between ^18^F-FDG PET scanning of AAAs and molecular characteristics (Table 2). Therefore, we included also studies without molecular characteristics available. While there are studies showing increased ^18^F-FDG uptake in patients with AAAs correlated with clinical events, there are also studies reporting decreased ^18^F-FDG uptake in AAA patients compared to controls. Literature suggests that ^18^F-FDG PET scanning might be useful in displaying molecular alterations characteristic of the weakening of the AAA wall. However, it still remains a question whether these molecular characteristics of aortic wall weakening might lead to better rupture and growth prediction. Moreover, most studies are limited by a very small patient population. Larger patient populations are warranted. Standardized imaging protocols and quantification methods are essential to compare patient populations. However, given the conflicting evidence to date with ^18^F-FDG PET scanning, it is unlikely that a reliable quantitative cutoff value to predict rupture risk may be established. The potential of change in quantitative measure on serial ^18^F-FDG PET scanning may be helpful. Moreover, animal studies in which non-invasively detection of inflammation or proteolysis in AAA wall by means of in vivo molecular imaging is investigated and needs to be implemented in humans in order to improve rupture risk stratification.

**Table 2:** Main findings of the systematic review

Finding	Reference
Increased FDG in AAA compared to controls	[13–15]
No difference or decreased FDG uptake in AAA compared to controls	[16–19]
Symptoms cause increased FDG uptake	[14]
Inflammatory AAAs have increased FDG uptake	[20]
FDG uptake correlates with pathological weakening of the aortic wall	[14, 25, 26, 29]
No correlation between FDG uptake and recent AAA growth	[14, 20]
Inverse correlation between FDG uptake and future expansion	[22, 23]
Inverse correlation between FDG uptake and aortic wall calcification	[16]
Positive association between FDG uptake and future AAA expansion or rupture	[11, 12, 21]

## Additional file

Additional file 1:
**The search query used in this study.** Medline, EMBASE and the Cochrane database were searched in september 2015 for relevant articles. After inclusion and exclusion, 18 relevant articles reporting on PET scanning of aortic aneurysms were included. (DOCX 20 kb)

## Abbreviations

^18^F-FDG: fludeoxyglucose F18
AAA: abdominal aortic aneurysm
CRP: C-reactive protein
CT: computed tomography
ECM: extracellular matrix
MRI: magnetic resonance imaging
PET: positron emission tomography
