# Supplementary material for: 18F-FDG PET scanning of abdominal aortic aneurysms and correlation with molecular characteristics: a systematic review
Source: EJNMMI Res. 2015 Dec 23;5:76. doi: 10.1186/s13550-015-0153-8 (PMC4688285; doi:10.1186/s13550-015-0153-8)
Supplement: Additional file 1: — The search query used in this study. Medline, EMBASE and the Cochrane database were searched in september 2015 for relevant articles. After inclusion and exclusion, 18 relevant articles reporting on PET scanning of aortic aneurysms were included. (DOCX 20 kb) [file 13550_2015_153_MOESM1_ESM.docx]

**Additional file 1**

The following search query was used in Medline:

((((Abdominal [TIAB] OR Thorac* [TIAB] OR thoracoabdominal [TIAB] OR Aortic [TIAB]) AND (aneurysm* [TIAB])) OR AAA [TIAB] OR TAA [TIAB]) AND (“Positron Emission Tomography” [tiab] OR PET [tiab] OR PET-CT [tiab] OR PET-MRI [tiab] OR “PET-imaging”[tiab])) OR ((((Abdominal [TIAB] OR Thorac* [TIAB] OR thoracoabdominal [TIAB] OR Aortic [TIAB]) AND (aneurysm* [TIAB])) OR AAA [TIAB] OR TAA [TIAB]) AND (“Positron Emission Tomography” [tiab] OR PET [tiab] OR PET-CT [tiab] OR PET-MRI [tiab] OR “PET-imaging”[tiab]) AND (plaque[tiab] OR histolog*[tiab] OR immunohisto*[tiab] OR patholog*[tiab] OR molecular [tiab] OR RNA[tiab] OR microarray[tiab] OR DNA [tiab] OR Protein [tiab]))

‘‘[TIAB]’’ is the abbreviation used for Title/ Abstract in Medline, and demands the presence of the preceding text in either the title or the abstract of the article. This search in Medline

generated 150 articles. The same search strategy was used in EMBASE (only ‘‘[TIAB]’’ had to be exchanged for ‘‘:ti,ab‘‘), rendering 121 articles. Medline and EMBASE search strategy yielded a total of 271 possibly relevant articles (figure 1). The Cochrane library was

manually searched, yielding no relevant articles.

((((Abdominal: ti,ab OR Thorac: ti,ab thoracoabdominal: ti,ab OR Aortic: ti,ab) AND (aneurysm*: ti,ab)) OR AAA: ti,ab OR TAA: ti,ab) AND (“Positron Emission Tomography” : ti,ab OR PET: ti,ab OR PET-CT: ti,ab OR PET-MRI: ti,ab OR “PET-imaging” : ti,ab)) OR ((((Abdominal: ti,ab OR Thorac*: ti,ab OR thoracoabdominal: ti,ab OR Aortic: ti,ab) AND (aneurysm*: ti,ab)) OR AAA: ti,ab OR TAA: ti,ab) AND (“Positron Emission Tomography” : ti,ab OR PET: ti,ab OR PET-CT: ti,ab OR PET-MRI: ti,ab OR “PET-imaging” : ti,ab) AND (plaque: ti,ab OR histolog*: ti,ab OR immunohisto*: ti,ab OR patholog*: ti,ab OR molecular: ti,ab OR RNA: ti,ab OR microarray: ti,ab OR DNA: ti,ab OR Protein: ti,ab))
